# Surgical Repair of Acetabular Fracture Using Unidirectional Porous *β*-Tricalcium Phosphate

**DOI:** 10.1155/2019/6860591

**Published:** 2019-04-16

**Authors:** Hiroshi Kumagai, Masashi Iwasashi, Toru Funayama, Satoshi Nakamura, Hiroshi Noguchi, Masao Koda, Masashi Yamazaki

**Affiliations:** ^1^Department of Orthopedics Surgery, Faculty of Medicine, University of Tsukuba, Japan; ^2^Department of Orthopedics Surgery, Tsukuba Medical Center Hospital, Tsukuba, Japan

## Abstract

We report a case of an acetabular fracture treated using a unidirectional porous *β*-tricalcium phosphate artificial bone (Affinos®) to surgically repair bone defects. An 82-year-old man sustained an acetabular fracture on the left side and presented with shock on arrival along with impaired vital signs and systolic blood pressure. Upon stabilization, we performed an open reduction and internal fixation. However, there were significant bone defects, which were then fixed using Affinos® (both blocks and granules), an artificial *β*-tricalcium phosphate bone with a porosity of 57% (pore size: 25–300 *μ*m), characterized by a novel unidirectional porous structure. By 18 months postoperatively, the patient was able to perform stair climbing and absorption and bone fusion around the artificial bone were observed. Affinos® has a frost-like structure, which endows it with good tissue-invasive properties because of the capillary effect. Moreover, it has excellent osteoconduction capability. In this case, both Affinos® blocks and granules showed good affinity, absorption, and bone substitution. Further prospective studies are required to confirm our findings.

## 1. Introduction

Bone grafting is necessary to treat complex fractures with significant bone defects [1, 2]. Common sources of autologous bone grafts are the iliac crest and fibula. Although it has its benefits, the graft harvest procedure also carries the disadvantage of introducing a second surgical site with potential for morbidity, including pain, infection, and neurovascular injury [3–5]. Therefore, various artificial bone substitutes have been developed as an alternative to autologous bone grafts and have been clinically applied in recent years [6, 7]. Artificial bone substitutes are a convenient and common option in Japan because of the scarcity of bone banking facilities [8]. In addition, sales of allograft bones are not permitted in Japan.

Affinos® (Kuraray Co., Tokyo, Japan), codeveloped with the Department of Orthopedics Surgery, Faculty of Medicine, University of Tsukuba, is a *β*-tricalcium phosphate (*β*-TCP) artificial bone with a porosity of 57% (pore size: 25–300 *μ*m) and consists of a novel unidirectional porous structure [9]. Using both high-strength and porous hydroxyapatite (HA) and *β*-TCP would have caused issues with early loading in a pelvic fracture. Moreover, Affinos® shows balanced artificial bone resorption and new bone formation and has been successfully used in clinical cases [10]. Therefore, we selected Affinos® to treat our case.

We report a case of an acetabular fracture treated using Affinos® to surgically repair bone defects and detail the outcomes.

## 2. Case Presentation

**Figure 1:**
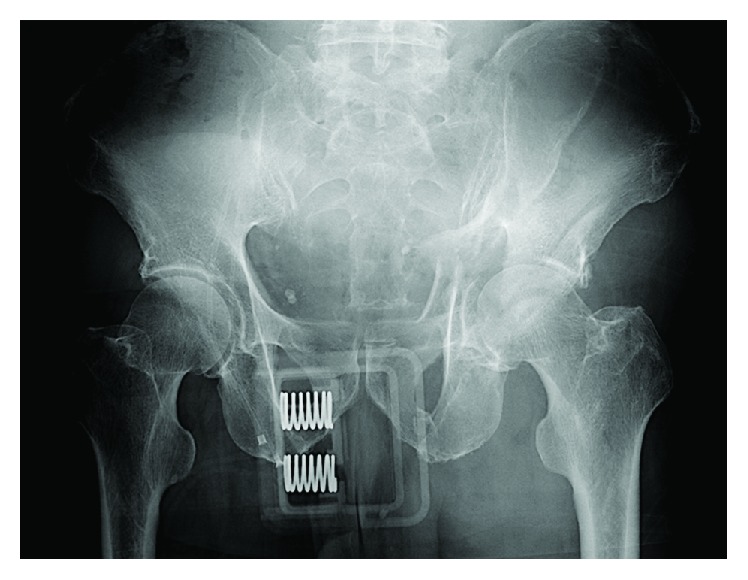
Initial radiographs of the fracture sites. An anteroposterior pelvic radiograph showing the fracture of both columns of the left acetabulum.

**Figure 2:**
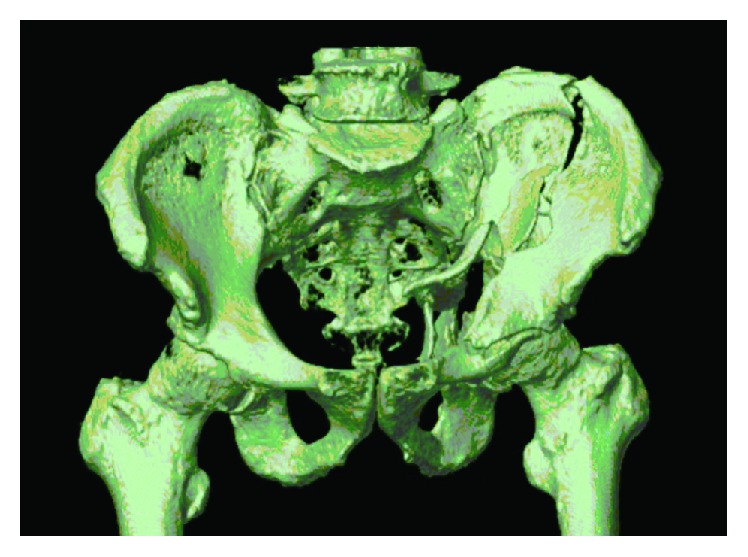
Three-dimensional computed tomography scan of the fracture showing displacement of both columns and impaction of the femoral head.

An 82-year-old man sustained an acetabular fracture on the left side involving the anterior and posterior columns after falling from a bicycle (Figures 1 and 2). He was in shock on arrival with impaired vital signs and systolic blood pressure. Contrast computed tomography scanning showed bleeding from the internal iliac artery. Hence, external fixation and transcatheter arterial embolization were performed in the emergency room on the same day. He then underwent internal fixation 11 days after the injury when his condition was stable. Surgical repair of the fracture was performed in two phases.

**Figure 3:**
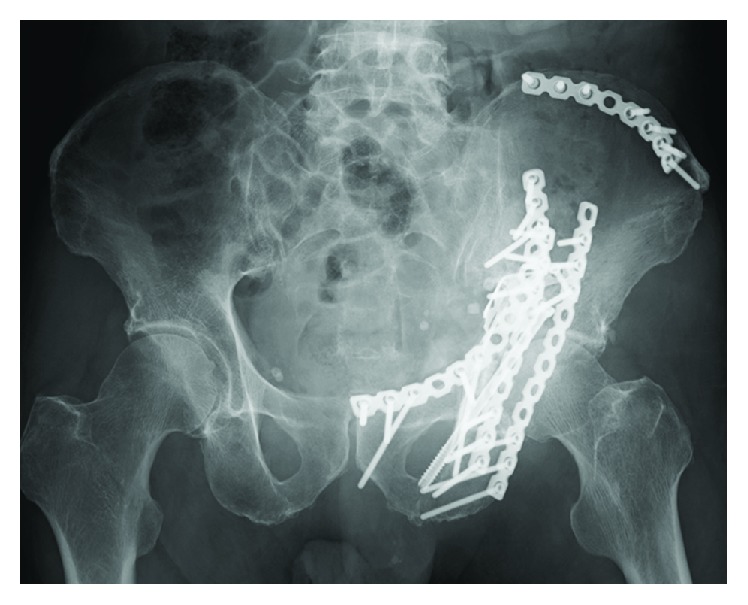
Postoperative radiographs. Both columns have been reduced and plated.

**Figure 4:**
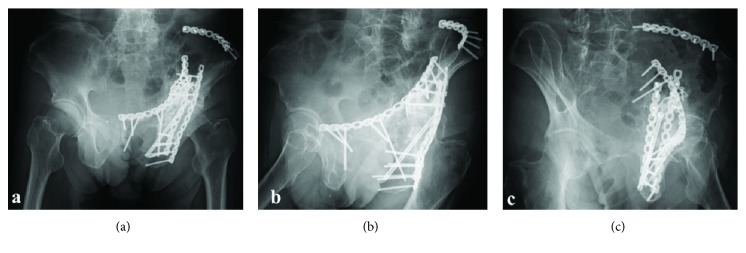
(a) Anteroposterior view and (b, c) Judet views of radiographs at the final follow-up, 18 months postoperatively.

**Figure 5:**
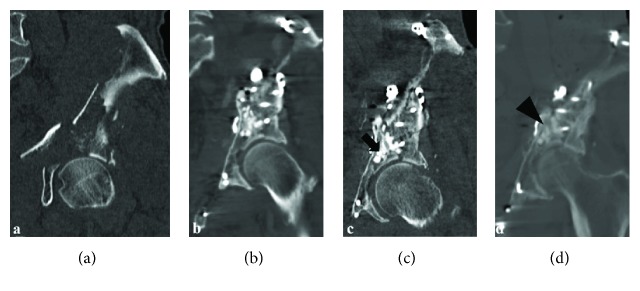
Pre- and postoperative coronal computed tomography scans. (a) There are extensive bone defects on the acetabular loading surface. (b) Immediately after surgery. (c) Three months after surgery: the scan shows that Affinos® transplanted into the bone defect area has not been absorbed (arrow). (d) Eighteen months after surgery: absorption and bone fusion around the artificial bone were observed (triangle).

First, we used the ilioinguinal approach to access the anterior column and carried out an open reduction and internal fixation (ORIF) using a reconstruction plate and screws. The displaced large fragment of the quadrilateral surface and arcuate line of the ilium were observed well. Although reduction was achieved, there were significant cancellous bone defects causing impaction of the acetabulum. Hence, Affinos®, in both granular and block forms, was placed in the bone defect without impaction so as to not break the micro structure before fixation with the plate. We then used the Kocher-Langenbeck approach to access the posterior column, wherein the overall fixation was neutralized by the reconstruction plate contoured to accommodate the shape of the posterior column (Figure 3). Partial weight bearing started 6 weeks after surgery, and the patient could walk 100 m using a cane 5 months postoperatively. At the time of the final follow-up, 18 months postoperatively, the patient was able to perform stair climbing without pain and the radiograph showed stable fixation without osteoarthritic change (Figure 4). The patient's modified Harris hip score was 85 at the final follow-up. We observed absorption and bone fusion around the artificial bone (Figure 5).

## 3. Discussion

Acetabular fractures often result from injuries of high-energy trauma, but 25% of fractures are also attributed to low-energy trauma like our current patient [11]. They are more common among elderly people; hence, bone vulnerability must be considered for treatment as per age [12, 13]. In acetabular fractures, the cancellous bone collapses because the femoral head impacts the acetabulum, and there is a tendency of bone loss after reduction. Total hip replacement surgery is indicated if reconstruction of the acetabular surface is difficult. Boudissa et al. reported that the mortality rate and perioperative infection rate were significantly higher in the total hip replacement group than in the ORIF group [11].

Transplantation using autologous bone, artificial bone, and allograft bone is useful for repairing bone defects of the acetabular loading surface [14]. However, there are not many bone banking facilities in Japan; therefore, autogenous bones or artificial bones are more commonly used [8]. Vedi et al. reported that the bone mineral density and thickness of the iliac cortical bone decline in elderly people [15]. There is also a report stating that the amount of progenitor cells contained in the iliac bone marrow declines with age [16]; hence, caution should be exercised when performing autogenous bone grafting in the elderly. Collecting the iliac bone from patients with an iliac fracture was reported rarely, and there were reports that bone grafting was performed by collecting autologous bones from the greater trochanter among such patients [17].

Artificial bone grafting has been used as a filling material for repairing acetabular defects in total hip arthroplasty [18, 19]; however, few cases using artificial bone for acetabular fractures were reported [14]. We used unidirectional porous *β*-TCP bone for the first time to treat the acetabular fracture.

In this case, we have used Affinos® to treat the bone defects on the acetabular loading surface. Affinos® has a frost-like structure similar to that of Regenos® (Kuraray Co., Tokyo, Japan), which endows it with good tissue invasion properties, because of capillary phenomena, and excellent osteoconduction capability [20, 21].

In this case, Affinos® blocks and granules showed good affinity, absorption, and bone substitution. Because Affinos® was not absorbed early, it was possible for bone formation to occur in order to substitute the acetabular loading surface.

It seems to represent a useful material for bone substitution and can be used successfully to repair bone defects in pelvic fractures extending to the acetabular loading surface. Prospective studies of adequate size are required to confirm our findings.
